# Color difference for shade determination with visual and instrumental methods: a systematic review and meta-analysis

**DOI:** 10.1186/s13643-023-02263-9

**Published:** 2023-06-08

**Authors:** Noha Morsy, Ahmed A. Holiel

**Affiliations:** Department of Conservative Dentistry, Alexandria University, Champlion St, Alexandria, Egypt

**Keywords:** Color difference, ΔΕ, Instrumental, Shade, Visual

## Abstract

**Background:**

Shade determination is a critical step for the fabrication of a satisfactory restoration. Visual shade selection with conventional shade guides is *subjective* and influenced by variables related to light, observer, and object. Shade selection devices have been introduced to provide subjective and quantitative shade values. This systematic review and meta-analysis aimed to compare the color difference for shade selection with visual and instrumental methods.

**Methods:**

An initial search was conducted on databases (MEDLINE via PubMed, Scopus, and Web of Science) in addition to a manual search through references of identified articles. Studies comparing the accuracy of visual and instrumental shade selection based on ΔΕ were included in data synthesis. Mean differences (MDs) and 95% confidence intervals (CIs) were calculated to estimate the effect size for global and subgroup meta-analysis using the inverse variance weighted method and random-effects model (*P* ˂ 0.05). Results were presented as forest plots.

**Results:**

The authors identified 1776 articles from the initial search. Seven in vivo studies were included in the qualitative analysis of which six studies were included in the meta-analysis. For the global meta-analysis, the pooled mean (95% CI) was − 1.10 (− 1.92, − 0.27). Test for overall effect showed that instrumental methods were significantly more accurate than visual methods with significantly less ΔΕ (*P* = 0.009). Test for subgroup difference showed that the type of instrumental shade selection method used had a significant effect on accuracy (*P* ˂ 0.001). Instrumental methods including spectrophotometer, digital camera, and smartphone showed significantly better accuracy compared with visual shade selection (*P* ˂ 0.05). The greatest mean difference was found between the smartphone and visual method with a mean (95% CI) of − 2.98 (− 3.37, − 2.59) with *P* ˂ 0.001 followed by digital camera and spectrophotometer. There was no significant difference in accuracy between IOS and visual shade selection (*P* = 1.00).

**Conclusions:**

Instrumental shade selection with a spectrophotometer, digital camera, and smartphone showed significantly better shade matching compared with a conventional shade guide, whereas IOS did not improve the shade matching significantly compared with shade guides.

**Review registration:**

PROSPERO CRD42022356545

**Supplementary Information:**

The online version contains supplementary material available at 10.1186/s13643-023-02263-9.

## Background

Tooth color determination is a critical and challenging step in restorative dentistry. Color determination can be performed by using visual methods or the more recently introduced instrumental methods. Visual methods employ dental shade guides which are subjective and can be affected by variables that may affect the results including the observer’s age, gender, skills, eye fatigue, and ambient light. However, the visual method is still the most common procedure for shade determination as shade guides are available, economic, and relatively easy to use [1, 2].

Instrumental methods for color determination have been developed to overcome the drawbacks of visual methods and provide objective color determination. These devices include spectrophotometers, intraoral scanners (IOSs), digital cameras, and smartphones. Spectrophotometer can measure the color by assessing the spectral reflectance or transmittance of the object and provides the CIELab values (Commission International del’Eclairage, L: the luminosity of the object, a: the chroma in the red-green axis, and b: the chroma in the yellow-blue axis). IOSs are recently equipped with a colorimeter and software to allow shade determination. This step can be performed as an integral part of the digital scanning. Digital photographs with smartphones and digital cameras are now a routine in many dental clinics for documentation purposes. In addition, these photographs can be used for shade determination by using image processing software or smartphone applications [2, 3].

CIE has introduced the color difference concept (ΔE), which describes the perceptibility and acceptability of the shade selected. Perceptibility is related to the discrimination between the color of the restoration and adjacent tooth while acceptability represents the acceptance of the restoration color. In the literature, a color difference is considered acceptable with ΔΕ ˂ 6.8. With regard to perceptibility, the human eye can detect a color difference when ΔΕ ≥ 3.7 intraorally and with ΔΕ ≥ 1 under standardized conditions [4–6].

In the literature, few studies have compared the visual and instrumental methods based on color differences (ΔΕ). Therefore, this systematic review was conducted to combine the available studies comparing visual and instrumental methods for shade determination. The PICO (P: population; I: intervention; C: control; O: outcome) question for this review was that for natural dentition (P), do instrumental methods (I) compared to visual methods (C) allow shade determination with less color difference (O)? The null hypothesis was that no significant difference would be found in ΔΕ between visual and instrumental methods.

## Methods

### Study design

This systematic review followed the guidelines of the Preferred Reporting Items for Systematic Reviews and Meta-Analyses (PRISMA) [7]. The protocol was registered on the international prospective register of systematic reviews (PROSPERO) with registration number CRD42022356545. The following questions were formulated and addressed in this review:

- Do instrumental shade selection using spectrophotometers, IOSs, digital cameras, and smartphones produce better shade matching compared to visual method using shade guides (based on ΔΕ)?
- Do the kind of instrumental shade selection method affect the accuracy of shade matching (based on ΔΕ)?

### Search strategy

An initial search was conducted on databases (MEDLINE via PubMed, Scopus, and Web of Science) in April 2022 by one author (M.N.) using the keywords presented in Additional file 1. In addition, a manual search was performed through the lists of references of identified records for additional eligible articles. The search was limited to English articles published in peer-reviewed journals between 2010 and 2022. Clinical or in vitro studies comparing visual and instrumental methods for shade selection in natural teeth and reporting ΔΕ as an outcome were eligible for the study. Two authors (M.N., H.A.) filtered the titles, the duplicates were removed manually, and the selected abstracts were filtered for identifying articles eligible for full-text reading and data synthesis. Any disagreement was resolved by discussion between the two reviewers. The references were managed using a spreadsheet (Microsoft Excel 2019 VL 16.44; Microsoft Corp., WA, USA). The included studies were analyzed by one author (M.N.) to extract data items summarized in Table 1.

**Table 1:** Characteristics of included studies

Author	Study type	Selection method	Sample size	ΔΕ (mean ± SD) µm	Conclusions
**Hampé-Kautz **[8]	In vivo	1. Visual method (Vita 3D-MASTER shade guide) 2. Instrumental method (Spectrophotometer) 3. Instrumental method (TRIOS III IOS) 4. Instrumental method (CEREC Omnicam IOS)	40	2.35 ± 0.25 1.85 ± 0.26 2.75 ± 0.27 2.75 ± 0.23	Shade determination with a spectrophotometer showed the best accuracy while IOSs presented the worst results
**Jorquera **[9]	In vivo	1. Visual method (Vita Classical shade guide) 2. Instrumental method (digital camera with a cross-polarized filter) 3. Instrumental method (Smartphone with light-correction filter)	15	5.32 ± 0.64 2.75 ± 0.40 2.34 ± 0.42	Shade selection with a digital camera and smartphone was significantly more accurate than the visual method
**Alshiddi **[10]	In vivo	1. Visual method (Vita 3D-Master shade guide) 2. Instrumental method (spectrophotometer)	8	4.22 ± 1.56 3.75 ± 1.71	Shade matching with the instrumental method is significantly more accurate than the visual method
**Czigola **[11]	In vivo	1. Visual method (Vita 3D-Master shade guide) 2. Visual method (Vita Classical shade guide) 3. Instrumental method (spectrophotometer) 4. Instrumental method (TRIOS III IOS)	10	NR	Vita 3D-Master shade guide produced the best shade matching followed by the spectrophotometer, IOS, and Vita Classical shade guide
**Mahn **[12]	In vivo	1. Visual method (Vita Classical shade guide) 2. Instrumental method (digital camera with a cross-polarized filter)	60	7 ± 5.14 6.05 ± 2.17	The instrumental shade selection with a digital camera had better shade selection acceptability compared with the visual method
**Alsaleh **[13]	In vivo	1. Visual method (Vita Classical shade guide) 2. Instrumental method (spectrophotometer)	15	5.85 ± 2.90 5 ± 2.50	The instrumental shade assessment had better acceptability than the visual assessment
**Brandt **[14]	In vivo	1. Visual method (Vita 3D-Master shade guide) 2. Instrumental method (TRIOS III IOS)	107	5.52 ± 2.47 4.99 ± 2.73	The shade determination with IOS is a good alternative to visual shade selection or can be used in conjugation with it

IOS, intraoral scanner; SD, standard deviation

### Meta-analysis

The included studies were assessed for risk of bias (ROB) by two authors (M.N., H.A.) using the Cochrane Collaboration tool for assessing the risk of bias in randomized trials [15]. A global and subgroup meta-analysis was performed for studies reporting the mean values of ΔΕ by using a review manager software program (RevMan 5.4.1; Cochrane Collab., London, UK). The effect size of each study and the overall effect size was calculated as mean differences (MDs) and 95% confidence intervals (CIs) by using the inverse variance weighted method and random-effects model (*P* ˂ 0.05). The *I*^2^ index was used to estimate heterogeneity with values greater than 50% and *P* < 0.1 considered as statistically significant heterogeneity [16]. Results were presented as forest plots.

## Results

**Fig. 1:**
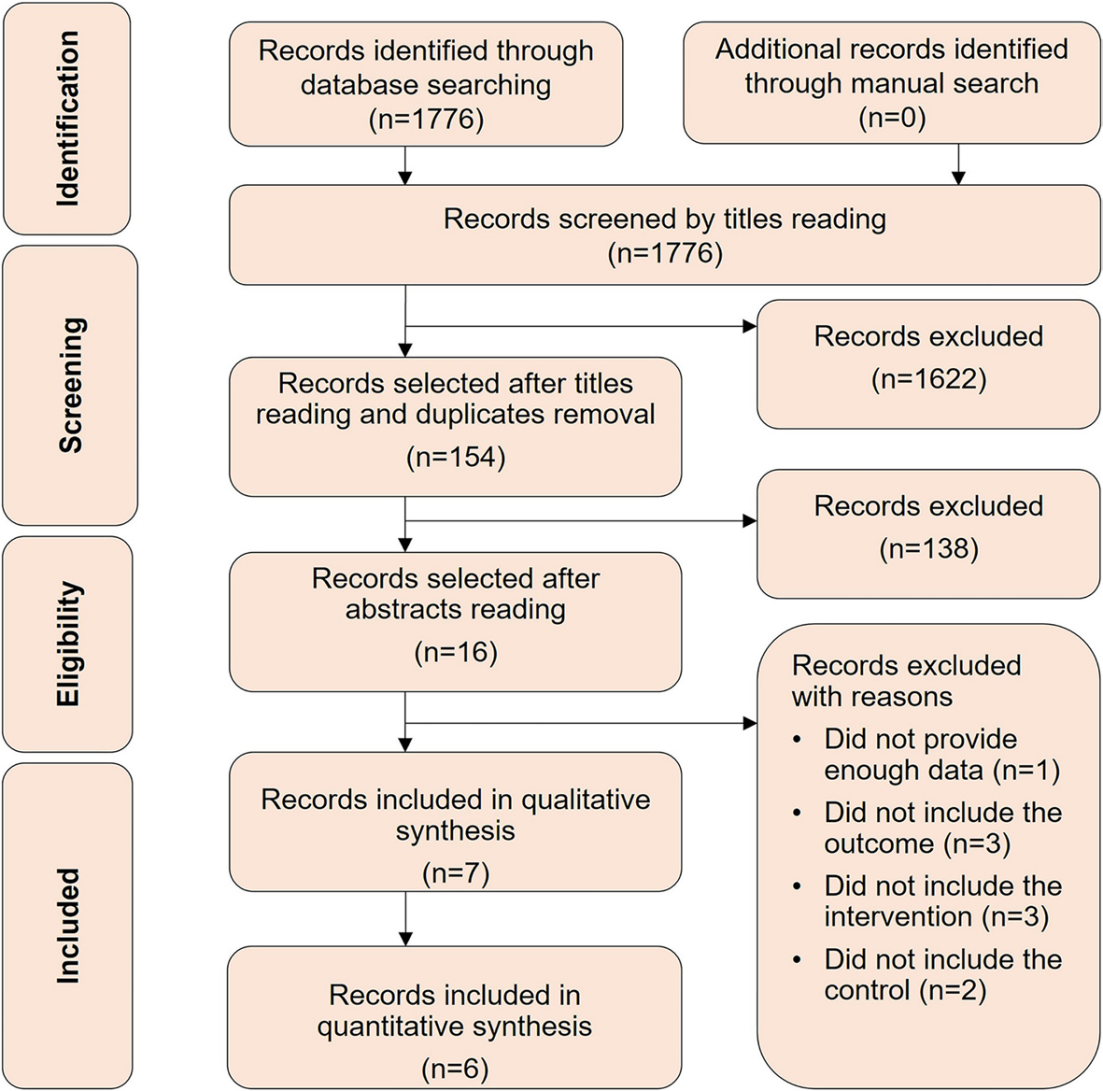
Preferred reporting items of systematic reviews and meta-analyses flow diagram for search strategy and study selection

The authors identified 1776 articles from the initial search. After screening titles and abstracts and duplicates removal, 16 articles were eligible for full-text reading. Seven studies [8–14] were included in qualitative analysis of which six studies [8–10, 12–14] were included in quantitative analysis. Figure 1 displays the results of the search strategy and study selection.

All included studies in data synthesis were in vivo including a total of 255 patients. The spectrophotometer was used for instrumental shade selection in four studies [8, 10, 11, 13]. IOSs were used in three studies [8, 11, 14], TRIOS IOS (3Shape Inc., Copenhagen, Denmark) was used in the three studies while one study [8] used both TRIOS and CEREC Omnicam IOS (Dentsply Sirona Inc., NC, USA). A digital camera was used in two studies [9, 12], and a smartphone was used in one study [9]. Four studies [8, 10, 11, 14] used the Vita 3D-Master shade guide (VITA Zahnfabrik, Bad Sackingen, Germany) for visual shade selection, and Vita Classical shade guide (VITA Zahnfabrik, Bad Sackingen, Germany) was used in four studies as well [9, 11–13]. In three studies [9, 12, 14], visual shade selection was conducted by a single experienced dentist. In one study [13], three clinicians with different levels of experience selected the shade visually. In another study [10], the visual shade selection was performed by a group of trained and another group of untrained students, they reported that training significantly improved the visual shade selection accuracy. One study [11] reported that the visual shade selection was conducted by trained students. Only one study [8] did not report the information about the operator who selected the shade visually. For the visual method, all included studies reported acceptable color differences below 6.8, and only one study [12] reported ΔΕ above the threshold value. However, six of the included studies [9–14] reported perceptible color differences above 3.7. For instrumental shade selection, all included studies reported acceptable color differences. In addition, four studies [10, 12–14] reported perceptible color differences.

Five studies [9, 10, 12–14] reported that instrumental shade selection is more accurate compared with the visual method. A study by Czigola et al. [11] reported better shade matching with the Vita 3D-Master shade guide compared with the spectrophotometer and IOS whereas Vita Classical shade guide produced less accuracy compared with both instrumental methods. Moreover, Hampé-Kautz et al. [8] reported better accuracy for the Vita 3D-Master shade guide compared with IOS while the shade guide had less favorable results compared with the spectrophotometer.

**Fig. 2:**
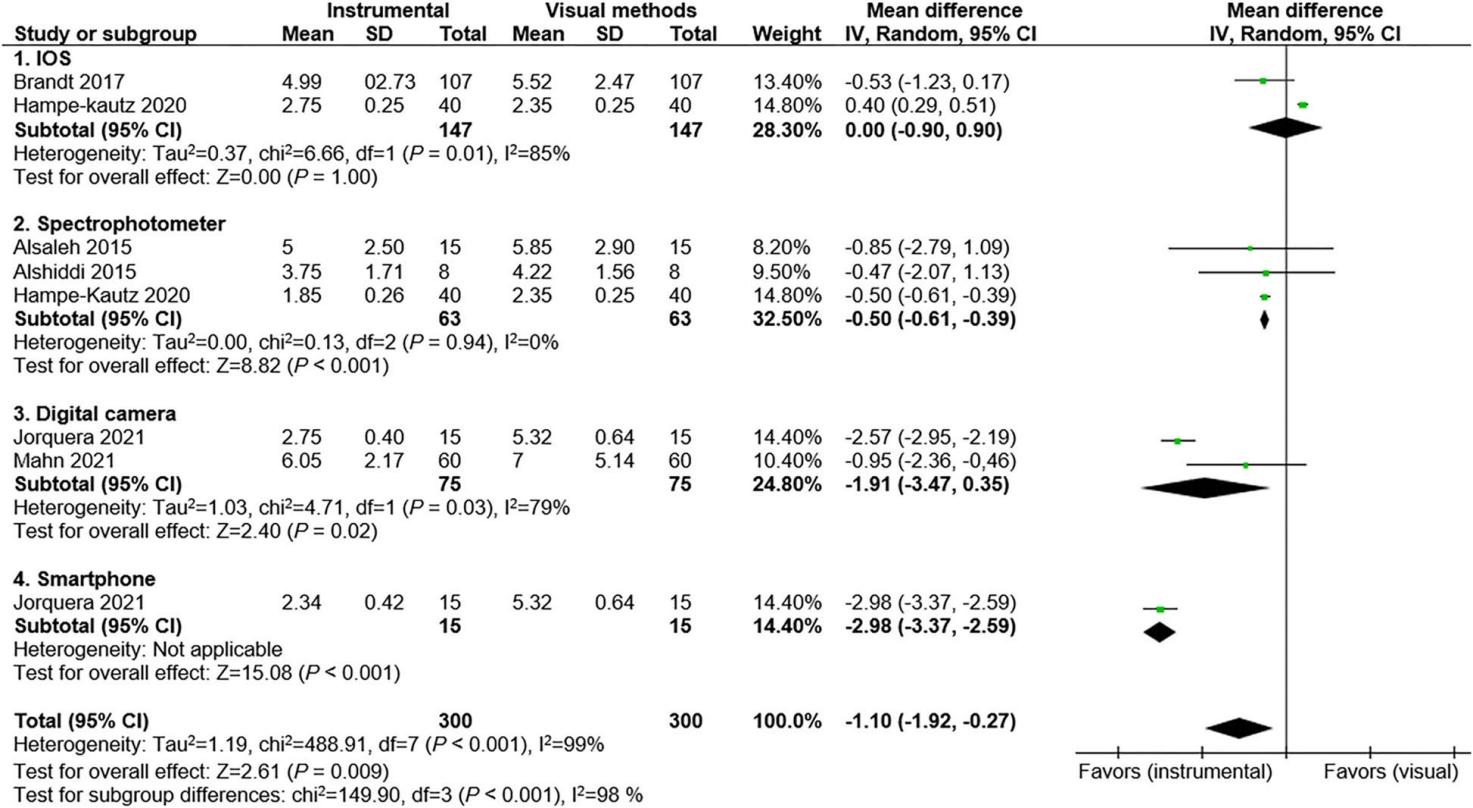
Forest plot for ΔΕ differences between visual and instrumental methods; CI confidence interval, SD standard deviation, IV inverse variance

A meta-analysis was performed for six studies [8–10, 12–14] as the study by Czigola et al. [11] did not report ΔΕ as the mean ± standard deviation (Fig. 2). The pooled mean (95% CI) was − 1.10 (− 1.92, − 0.27). Test for overall effect showed that instrumental methods are significantly more accurate than visual methods with significantly less ΔΕ (*P* = 0.009). Test for subgroup difference showed that the type of instrumental shade selection method used has a significant effect on accuracy (*P* ˂ 0.001). Instrumental methods including spectrophotometer, digital camera, and smartphone showed significantly better accuracy compared with visual shade selection (*P* ˂ 0.05). The greatest mean difference was found between the smartphone and visual method with a mean (95% CI) of − 2.98 (− 3.37, − 2.59) with *P* 0 ˂ 0.001 followed by digital camera and spectrophotometer. There was no significant difference in accuracy between IOS and visual shade selection (*P* = 1.00).

The heterogeneity was high for all included studies in the quantitative synthesis except for the studies comparing spectrophotometer and visual method. This might be due to the variability in the study setup. All included studies had an overall low ROB. However, six studies had unclear ROB concerning blinding of outcome assessment (detection bias) as the visual shade selection was conducted in these studies without masking the shade guide samples. The assessment results are presented in Table 2.

**Table 2:** Evaluation of the risk of bias

Study	Random sequence generation (selection bias)	Allocation concealment (selection bias)	Blinding of participants and researchers (performance bias)	Blinding of outcome assessment (detection bias)	Incomplete outcome data (attrition bias)	Selective reporting (reporting bias)	Other bias
**Hampé-Kautz **[8]	Low	Low	Low	Unclear	Low	Low	Low
**Jorquera **[9]	Low	Low	Low	Unclear	Low	Low	Low
**Alshiddi **[10]	Low	Low	Low	Unclear	Low	Low	Low
**Czigola **[11]	Low	Low	Low	Low	Low	Low	Low
**Mahn **[12]	Low	Low	Low	Unclear	Low	Low	Low
**Alsaleh **[13]	Low	Low	Low	Unclear	Low	Low	Low
**Brandt **[14]	Low	Low	Low	Unclear	Low	Low	Low

## Discussion

This review aimed to compare the acceptability and perceptibility of shade selection with instrumental and visual methods. The null hypothesis was rejected as the instrumental methods presented significantly less ΔΕ compared with visual methods indicating a significantly better shade matching. For both visual and instrumental methods, the color difference had adequate acceptability. However, the color difference was perceptible in most of the included studies for the instrumental method and almost all studies for the visual method.

Visual shade matching is a subjective method affected by three main factors including the light source, object properties, and observer. The ideal light for shade selection should have a well-distributed wavelengths spectrum between 400 and 700 nm, and this light should have a color temperature of 5500 Kelvin and a color-rendering index greater than 93 (the color-rendering index is the ability of the light source to disclose the colors of different objects correctly in comparison with an ideal or natural light source). An ideal light condition can be rarely obtained in clinics [17]. The object properties including dehydration and contrast with the surrounding teeth, lips, and gingiva can affect the shade of the object. In addition, the observer’s shade matching ability may be affected by age, gender, skills, and color perception disorders [2]. It is also worth mentioning that the available shade guides do not represent the true teeth color which also may explain the results obtained in this study [18]. On the other side, shade-matching devices eliminate the variables associated with visual matching to improve accuracy and provide quantitative data about shade. Spectrophotometer is equipped with a light source with a wavelength between 400 and 700 nm and some types of spectrophotometers can isolate the ambient light with a special mouthpiece and reduce metamerism with a specially designed probe [2]. Digital cameras and smartphones are supplied with cross-polarized filters for color correction of light and reduction of specular reflection from glossy surfaces and flash photography which improves the photograph quality for shade matching [9, 12].

The subgroup analysis showed that the spectrophotometer, digital camera, and smartphone had significantly better accuracy compared with visual shade determination while IOS did not improve the shade-matching accuracy. Shade selection with IOS depends on the acquisition process applied by the scanner where the light emission and collection by a sensor can affect the results. Consequently, the shade selection with IOS is more sensitive than the spectrophotometer to manipulation errors, type of IOS, and poor color analysis software within the IOS. Moreover, the spectrophotometer focuses on a small spot and is placed in close contact with the surface while the IOS scans a wider area with more susceptibility to errors. In addition, IOS can be affected by ambient light in contrast to the spectrophotometer [2, 19].

The findings of this review and meta-analysis agree with a similar study by Hardan et al. [3] who reported better shade matching for spectrophotometer and digital photography compared with visual methods.

The limitations of this research are the few studies included in the analysis and the substantial heterogeneity for all included studies in the quantitative synthesis except for the studies comparing spectrophotometer and visual method. In this study, the search was limited to English articles published in peer-reviewed which may have limited the number of included studies. The significant heterogeneity in this research was not investigated by a meta-regression or a sensitivity test because only a few studies were included in the analysis.

From a practical perspective, smartphones and digital cameras are routinely used for documentation photographs in dental clinics and the findings of the current research encourage clinicians to use such photographs for shade selection which is also considered as cost-effective, fast, easy, and accurate shade selection method. However, the results of this study should be interpreted with caution as few studies were included in data synthesis; therefore, further research is recommended to compare visual shade selection with the available shade selection devices.

## Conclusion

Based on the findings of this systematic review and meta-analysis, the following conclusions were drawn:

1. Shade selection devices including the spectrophotometer, smartphone, and digital camera can significantly improve the accuracy of shade matching compared with visual shade selection with shade guides.
2. Shade selection accuracy with IOSs did not differ significantly from visual methods.
3. Further studies are needed to compare the color difference for shade matching with visual and instrumental methods.

## Supplementary Information

**Additional file 1.** 

## Data Availability

The datasets used and/or analyzed during the current study are available from the corresponding author on reasonable request.

## Abbreviations

CI: Confidence interval
CIELab: Commission International del’Eclairage, L: the luminosity of the object, a: the chroma in the red-green axis, b: the chroma in the yellow-blue axis
3D: 3-Dimensional
IOS: Intraoral scanner
MD: Mean difference
PICO: P: population, I: intervention, C: control, O: outcome
PRISMA: Preferred Reporting Items for Systematic Reviews and Meta-Analyses
ROB: Risk of bias
SD: Standard deviation
