# Supplementary material for: Color difference for shade determination with visual and instrumental methods: a systematic review and meta-analysis
Source: Syst Rev. 2023 Jun 8;12:95. doi: 10.1186/s13643-023-02263-9 (PMC10249324; doi:10.1186/s13643-023-02263-9)
Supplement: Supplementary file 1 — Additional file 1. [file 13643_2023_2263_MOESM1_ESM.docx]

Search terms:

((digital OR scanner OR IOS OR instrument OR automatic OR spectrophotometer OR colorimeter OR colorimetry OR spectrophotometry OR photograph) AND (dent* OR teeth OR tooth OR enamel) AND (''color AND selection'' OR ''color AND perception'' OR ''color AND determination'' OR ''color AND measurement'' OR color OR ''color AND assessment'' OR shade OR ''shade AND selection'' OR ''shade AND determination'' OR ''shade AND measurement'' OR ''shade AND assessment'' OR ''shade AND perception'' OR ''shade AND guide'')).
